# Dual Role of Lysophosphatidic Acid Receptor 2 (LPA_2_) in Amyotrophic Lateral Sclerosis

**DOI:** 10.3389/fncel.2021.600872

**Published:** 2021-03-25

**Authors:** Maria Puigdomenech-Poch, Anna Martínez-Muriana, Pol Andrés-Benito, Isidre Ferrer, Jerold Chun, Rubèn López-Vales

**Affiliations:** ^1^Departament de Biologia Cellular, Fisiologia i Immunologia, Institut de Neurociències, Centro de Investigación Biomédica en Red sobre Enfermedades Neurodegenerativas (CIBERNED), Universitat Autònoma de Barcelona, Bellaterra, Spain; ^2^Centro de Investigación Biomédica en Red de Enfermedades Neurodegenerativas (CIBERNED), Madrid, Spain; ^3^Departament de Patologia i Terapèutica Experimental, Hospital Universitari de Bellvitge, IDIBELL, L’Hospitalet de Llobregat, Universitat de Barcelona, Barcelona, Spain; ^4^Sanford Burnham Prebys Medical Discovery Institute, La Jolla, CA, United States

**Keywords:** amyotrophic lateral sclerosis, LPA_2_, lysophosphatidic acid, lysophosphatidic acid receptor, inflammation, motoneuron, muscle atrophy

## Abstract

Lysophosphatidic acid (LPA) is a pleiotropic extracellular lipid mediator with many physiological functions that signal through six known G protein-coupled receptors (LPA_1–6_). In the central nervous system (CNS), LPA mediates a wide range of effects including neural progenitor cell physiology, neuronal cell death, axonal retraction, and inflammation. Since inflammation is a hallmark of most neurological conditions, we hypothesized that LPA could be involved in the physiopathology of amyotrophic lateral sclerosis (ALS). We found that LPA_2_ RNA was upregulated in post-mortem spinal cord samples of ALS patients and in the sciatic nerve and skeletal muscle of SOD1^G93A^ mouse, the most widely used ALS mouse model. To assess the contribution of LPA_2_ to ALS, we generated a SOD1^G93A^ mouse that was deficient in *Lpar2*. This animal revealed that LPA_2_ signaling accelerates disease onset and neurological decline but, unexpectedly, extended the lifespan. To gain insights into the early harmful actions of LPA_2_ in ALS, we studied the effects of this receptor in the spinal cord, peripheral nerve, and skeletal muscle of ALS mice. We found that LPA_2_ gene deletion increased microglial activation but did not contribute to motoneuron death, astrogliosis, degeneration, and demyelination of motor axons. However, we observed that *Lpar2* deficiency protected against muscle atrophy. Moreover, we also found the deletion of *Lpar2* reduced the invasion of macrophages into the skeletal muscle of SOD1^G93A^ mice, linking LPA_2_ signaling with muscle inflammation and atrophy in ALS. Overall, these results suggest for the first time that LPA_2_ contributes to ALS, and its genetic deletion results in protective actions at the early stages of the disease but shortens survival thereafter.

## Introduction

Amyotrophic lateral sclerosis (ALS) is a fatal neurodegenerative disease caused by the progressive degeneration of motoneurons in the spinal cord and the brain. ALS leads to muscle weakness, spasticity, paralysis, and eventually the death of the patients within 2–5 years of clinical onset in the majority of the cases. At present, therapy is mainly symptomatic and fails to halt disease progression (Philips and Robberecht, 2011; Robberecht and Philips, 2013; Hardiman et al., 2017) ALS is inherited in 5–10% of cases but approximately 90–95% of ALS patients have no apparent family history and are often termed “sporadic ALS” (sALS; Philips and Robberecht, 2011; Robberecht and Philips, 2013). Nevertheless, they all share similar pathological features and are clinically indistinguishable.

A common feature of ALS and other neurological disorders is the occurrence of an inflammatory response in the central nervous system (CNS), peripheral nervous system (PNS), and muscles (Nagai et al., 2007; Yamanaka et al., 2008; Frakes et al., 2014; De Virgilio et al., 2016; Newcombe et al., 2018; Bright et al., 2019). Although immune cells play a crucial role in protecting host tissues from infection and help at maintaining homeostasis, immune cells also secrete various cytotoxic mediators that promote damage in healthy neighboring cells and cause cell death (López-Vales and David, 2012; Gilhus and Deuschl, 2019) This double-edged sword of inflammation likely depends on regulatory mediators that are present in the extracellular milieu. In neurological conditions, including ALS, an inflammatory response is believed to trigger more hazardous than protective actions, and thus, attenuating inflammation could reduce axonal damage and motor neuron degeneration in ALS (Appel et al., 2010; Philips and Robberecht, 2011; Robberecht and Philips, 2013).

Several studies have revealed the importance of lysophosphatidic acid (LPA) in modulating inflammation, including in the central nervous system (CNS; Choi et al., 2010; Choi and Chun, 2013; Yung et al., 2014, 2015). LPA is a lysophospholipid that is a potent lipid-signaling molecule with many different actions in the CNS including neural development (Hecht et al., 1996), axonal retraction (Bräuer et al., 2003; Broggini et al., 2010), neuronal death (Kingsbury et al., 2003; Steiner et al., 2006; Ramesh et al., 2018; Zhang et al., 2020), and inflammation (Kwon et al., 2018; Plastira et al., 2020). Moreover, LPA signaling has been related to various neurological disorders including psychiatric diseases (Mirendil et al., 2015; Yamada et al., 2015; Schneider et al., 2018; Tabbai et al., 2019), Alzheimer’s disease (Hwang et al., 2012; Shi et al., 2013; Ramesh et al., 2018) spinal cord injury (Goldshmit et al., 2012; Santos-Nogueira et al., 2015; López-Serrano et al., 2019), and the appearance of neuropathic pain responses (Lin et al., 2012; Ueda et al., 2013, 2018; González-Gil et al., 2020).

LPA mediates all these effects by signaling through 6 G protein-coupled receptors (LPA_1_–LPA_6_; Chun et al., 2013; Kihara et al., 2014). These receptors are expressed in almost all cells of the CNS and peripheral tissues (Choi et al., 2010; Choi and Chun, 2013; Yung et al., 2014, 2015). We have previously demonstrated that LPA_1_ and LPA_2_ contribute to the physiopathology of spinal cord injury by modulating microglial activation (Santos-Nogueira et al., 2015; López-Serrano et al., 2019). However, whether LPA exerts harmful or beneficial actions in ALS is currently unknown.

In the present work, we provide evidence for the first time that LPA contributes to ALS pathology. In particular, we found that the lack of LPA_2_ signaling reduces macrophage infiltration into the muscle and muscle atrophy in ALS animals, and slows disease onset and neurological deficits. However, the lack of this receptor paradoxically shortens the survival of SOD1^G93A^ mice.

## Materials and Methods

### Human Samples

Human samples obtained from ALS patients and controls were gently provided by the Institute of Neuropathology HUB-ICO-IDIBELL Biobank. The post-mortem interval between death and tissue processing was between 2 and 17 h. Necropsy samples of the spinal cord from human ALS patients (*n* = 22) and age-matched controls (*n* = 17) were removed and kept at −80°C or fixed by immersion in 4% buffered formalin. The lumbar anterior spinal cord was dissected frozen on a plate over dry ice using a binocular microscope at a magnification ×4. Cases with frontotemporal dementia were not included in the present series. Patients with associated pathology including Parkinson’s disease, Alzheimer’s disease (excepting neurofibrillary 103 tangle pathology stages I–II of Braak and Braak), tauopathies, vascular diseases, neoplastic diseases affecting the nervous system, metabolic syndrome, hypoxia, and prolonged axonal states such as those occurring in intensive care units were excluded. Cases with infectious, inflammatory, and autoimmune diseases, either systemic or limited to the nervous system were not included. Age-matched control cases had not suffered from neurologic or psychiatric diseases and did not have abnormalities in the neuropathologic examination, excepting sporadic neurofibrillary tangle pathology stages I–II of Braak and Braak. No C9ORF72, SOD1, TARDBP, and FUS mutations occurred in any case.

### Animals

SOD1^G93A^ mice [B6-Tg (SOD1-G93A)1Gur] were purchased from Jackson Laboratory (Bar Harbor, ME, USA). SOD1^G93A^ mice were heterozygous for the mutant SOD1 gene (Bright et al., 2019). This experimental model replicates the most relevant phenotypical and histopathological features of the human disease (Turner and Talbot, 2008). *Lpar2* deficient mice (mixed BL6/SVJ background) were provided by Dr. Jerold Chun (Contos et al., 2002). *Lpar2* null mice and wild-type littermates were crossed with SOD1^G93A^ mice to generate double transgenic mice that express mutant SOD1^G93A^ in heterozygosis but are homozygous knockout for *Lpar2* (*Lpar2*^−/−^). A total of 215 mice were used in this study. All mice used in the studies were housed in the Universitat Autònoma de Barcelona animal facilities, in standard cages and feed *ad libitum* with a light-dark cycle of 12 h.

### Rotarod and Survival

Rotarod test was done to assess motor deficits and define the disease onset in SOD1^G93A^ mice (Miana-Mena et al., 2005). Mice were placed onto the rotarod at a constant speed of 14 revolutions per minute. Animals were trained before the beginning of the experiment. 180 s was chosen as the arbitrary cut-off time. Rotarod test was then performed weekly from 8 to 20 or 22 weeks of age at the same speed. The time for which each animal could remain on the rotating cylinder was measured. Each animal was given three trials and the longest latency without falling was recorded.

The time of disease onset was defined at the time when mice could not remain in the rotarod for at least 180 s. The endpoint was determined when mice were not able to right themselves within 30 s of being placed on their side. At the endpoint, mice were euthanized by an overdose of pentobarbital sodium (Dolethal, Vetoquinol) following the requirements of the Animal Experimentation Ethical Committee of the Universitat Autònoma de Barcelona.

### Electrophysiological Test

#### Compound Muscle Action Potential (CMAP)

Animals were anesthetized with pentobarbital sodium (50 mg/kg, i.p, Sigma) and placed prone over a heating pad that maintains body temperature. Needle electrodes were placed deep into the sciatic nerve notch and the sciatic nerve was stimulated using single pulses of 0.02 ms duration (Grass S88) Recording microneedles were inserted superficially in the tibial anterior muscle. The recording needles were placed using a magnifier lens and guided by anatomical landmarks, to ensure reproducibility of needle location on all animals. Reference and ground electrodes were inserted at the third toe and the base of the paw, respectively. CMAPs were recorded from the tibialis *anterior* and gastrocnemius muscle. Signals were bandpass filtered (3 Hz to 3 kHz), amplified 100× for gastrocnemius and tibialis (P511AC amplifiers, Grass), and digitized with a Power Lab recording system (PowerLab16SP, ADInstruments) at 20 kHz. The amplitude of the M wave was studied from the difference between the baseline to the maximal negative peak.

#### Motor Unit Number Estimation (MUNEs)

Animals were anesthetized as described above. Electrodes were placed in the same place as for the CMAP registers and the amplitude for the M wave was recorded. For the MUNE assessment, the protocol used consists of the incremental technique. Starting from the subthreshold intensity, the sciatic nerves were stimulated with single pulses of gradually increased intensity until the first response appeared, representing the first motor unit recruited. With the next stimuli, quantal increases in the response were recorded. The data were represented as the frequency distribution of the single motor unit size (SU); as the mean of consistent increases (increments >100 μV) single motor unit potential (SMUP) and finally, MUNE results from the CMAP maximal amplitude divided by the average SMUP.

### Sciatic Nerve Injury

*Lpar2*^−/−^ mice and wild-type littermates were deeply anesthetized with a mixture of ketamine (90 mg/kg i.m.; Imagen, Merial) and xylazine (10 mg/kg i.m.; Rompun, Bayer). The right hindlimb was shaved and disinfected, and then, an incision in the skin and muscles was done to expose the sciatic nerve. The sciatic nerve was cut above the trifurcation and ligated to prevent axon regeneration and muscle reinnervation. The wound was closed and disinfected, and the mice were kept in a warm environment until complete recovery from anesthesia. Buprenorphine (0.01 mg/kg i.p.; Buprex, Indivior) was administrated intraperitoneally, twice a day, for 3 days.

### Quantitative Real-Time PCR (qPCR)

SOD1^G93A^ mice were transcardially perfused with sterile saline at 8 (pre-symptomatic), 12 (disease onset), 16 (symptomatic), and 20 weeks of age (late-symptomatic), and lumbar spinal cord, sciatic nerve, and gastrocnemius muscle were harvested and immediately frozen in liquid nitrogen. The same tissues were also harvested from WT littermates at 20 weeks of age. Samples were homogenized in QIAzol lysis reagent (Qiagen) using a tissue rupture, and RNA was isolated using RNeasy Lipid Tissue kit (Qiagen), according to the manufacturer’s protocol. One microgram of RNA was retro-transcribed using the Omniscript RT kit (Qiagen). Quantitative PCRs (qPCRs) were done using Brilliant III Ultra-Fast SYBR (Bio-Rad) and custom-designed primers. Primer sequences were the following *Lpar2* forward 5′-CTCACTGGTCAATGCAGTGGTATAT-3′, *Lpar2* reverse 5′-GAAGGCGGCGGAAGGT-3′. Glyceraldehyde 3-phosphate dehydrogenase (GAPDH); *Gapdh* forward 5′-TCAACAGCAACTCCCACTCTTCCA-3′, *Gapdh* reverse 5′-ACCCTGTTGCTGTAGCCGTATTCA-3′.

RNA from human spinal cord samples was isolated using RNeasy Mini Kit (Qiagen) and retro-transcribed using cDNA Reverse Transcription kit (Applied Biosystems, Foster City, CA, USA) following manufacturers’ kit instructions. qPCR was done using TaqMan-designed primers (Thermo Fisher Scientific, MA, USA) for gene expression of *LPA_1_* (Hs00173500_m1), *LPA_2_* (Hs01109356_m1), and HPRT1 (Hs02800695_m1).

Mouse and human qPCR data were analyzed using the double-delta cycle threshold (ΔΔCt) method on a MyiQ Single-Color Real-Time PCR Detection System (Bio-Rad). *Gapdh* and *HRPT1* were used as housekeeping genes for mouse and human samples, respectively.

### Histology

SOD1^G93A^ *Lpar2*^+/+^, SOD1^G93A^-*Lpar2*^−/−^, *Lpar2* null mice and wild-type littermates were euthanized at 16 weeks of age with an overdose of pentobarbital sodium (Dolethal, Vetoquinol) and transcardially perfused with 4% paraformaldehyde in 0.1 M phosphate buffer (PB). Also, *Lpar2* deficient mice and wildtype littermates with sciatic nerve injury were perfused at 3 weeks after the lesion. Samples were post-fixed in 4% PFA for 2 h and cryoprotected with 30% sucrose in 0.1 M PB at 4°C for 48 h. The spinal cords and muscles were fast-frozen at -20°C in a cryo embedding compound (Tissue-Tek OCT, Sakura), cut on a cryostat (Leica), and serially picked up in gelatine coated slides. The ventral root of the spinal nerve (L4) that fixed into 3% glutaraldehyde, 3% PFA in 0.1 PB at 4°C for 1 week, immersed in 2% osmium tetroxide in 0.1 M PB for 2 h, dehydrated through ethanol series and embedded in Epon resin. Semithin sections (0.5 μm thick) were cut by an ultramicrotome (Leica).

To analyze the preservation of motoneurons, transversal sections of the spinal cord (L4 and L5) were placed in a hotplate for 30 min, rehydrated, and stained in cresyl violet. The number of motoneurons was manually quantified in both ventral horns. To ensure reproducibility, only those neurons that were within the laminae IX, were larger than 20 μm and had a polygonal shape and prominent nucleoli were counted.

For immunofluorescence, spinal cord and muscle samples were placed on a hotplate at 37°C for 30 min, then rehydrated in 0.1 PBS for 5 min and blocked with a blocking buffer of 5% normal donkey serum (NDS) in 0.3% triton-PBS for 1 h at RT. The sections were incubated overnight at 4°C with primary antibodies against Iba1 (for microglia and macrophages; 1:500; Abcam), CD68 (1:100; Abcam), GFAP (for astrocytes; 1:500; Millipore). The sections were then washed in 0.3% triton-PBS and further incubated for 1 h at RT with the specific secondary antibodies tagged to an Alexa-594 or Alexa-488 fluorochrome (1:200, Invitrogen). After washing several times in 0.3% Triton-PBS and PBS, slides were incubated for 1 min in a solution containing DAPI (1 μg/ml, Sigma).

Microgliosis and astrogliosis were analyzed by quantifying the integrated density of Iba, CD68, and GFAP immunostaining at the spinal cord ventral horns of L4–L5 segments. Macrophage counts in the gastrocnemius were done by manually quantifying the number Iba1 and CD68 positive cells.

To analyze the muscle fiber area, cryostat transversal sections of gastrocnemius muscle were hydrated in water and stained for hematoxylin (Fluka, Sigma) and eosin (Merck, Millipore) for 5 min. The sections were dehydrated with series of graded ethanol rinses and mounted with DPX (Fluka).

To study motor axon preservation, ventral root (L4) semithin sections, were stained with 1% Borax (Sigma) and 1% Toluidine Blue (Fluka) in distillate water. The number of spared motor axons, with intact myelin sheaths and those with some degree of myelin damage were counted.

Samples were visualized with an Olympus BX51 microscope and the images were captured with Olympus DP50 digital camera. All Histological quantifications were done using ImageJ software (NIH, Bethesda, MD, USA).

### Statistical Analysis

Data are shown as mean ± standard error of the mean (SEM). Rotarod test results were analyzed using two-way repeated-measures ANOVA with *post-hoc* Bonferroni’s for multiple comparisons. Disease onset and the survival data were analyzed using the Mantel–Cox test. Gene expression, histological analysis, and electrophysiological results were analyzed by using one-way ANOVA with *post-hoc* Bonferroni’s for multiple comparisons of unpaired *t*-test. Statistical significance was considered at *p* < 0.05.

## Results

### LPA_2_ Transcripts Are Increased in Human and Mouse ALS Samples

**Figure 1:**
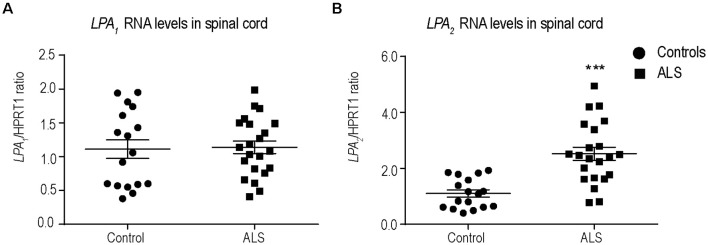
Characterization of Lysophosphatidic acid 1 (*LPA_1_*) and *LPA_2_* expression in the spinal cord of sporadic amyotrophic lateral sclerosis (sALS) patients. **(A,B)** Plots showing RNA levels of *LPA_1_* **(A)** and *LPA_2_* **(B)** in the spinal cord of sALS patients (*n* = 22) and controls (*n* = 17). Unpaired *t*-test. Data are shown as mean ± SEM. ***p < 0.001.

LPA exerts its biological effects by binding to 6 G protein-coupled receptors, known as LPA_1–6_. We have recently demonstrated that among the different LPA receptors, LPA_1_ (Santos-Nogueira et al., 2015) and LPA_2_ (López-Serrano et al., 2019) mediate demyelination and functional impairments in spinal cord injury. Therefore, we first examined the RNA levels of *LPA_1_* and *LPA_2_* in postmortem spinal cord samples of sALS patients and age-matched controls. *LPA_1_* and *LPA_2_* transcripts were found in the spinal cord of control and sALS individuals (Figures 1A,B). However, only the levels of *LPA_2_* significantly increased in sALS samples (Figures 1A,B).

**Figure 2:**
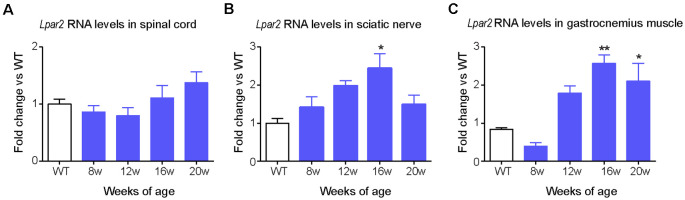
Dynamic changes in *Lpa*_2_ expression in SOD1^G93A^ mice. **(A–C)** Graphs showing the changes in *Lpar2* RNA levels in the spinal cord **(A)**, sciatic nerve **(B)**, and gastrocnemius muscle **(C)** of SOD1^G93A^ at 8, 12, 16, and 20 weeks of age relative to wild-type littermate mice. **(A)** *n* = 5 per group and time point. One-way ANOVA with Bonferroni’s *post hoc* test. *p < 0.05, **p < 0.01 vs. WT mice. Data are shown as mean ± SEM.

Based on these data, we focused our interest on LPA_2_ and characterized its expression in the spinal cord, sciatic nerve, and muscle of SOD1^G93A^ mice. QPCR revealed that the expression of *Lpar2* in these three tissues was similar in SOD1^G93A^ mice at pre-symptomatic stages of the disease (8 weeks of age) than in wild-type littermates (Figures 2A–C). RNA levels of *Lpar2* in the spinal cord of SOD1^G93A^ mice tended to increase once they show marked clinical symptoms of the disease (16–20 weeks of age), although this result did not reach statistical significance (Figure 2A). The transcripts of *Lpar2* in the sciatic nerve and gastrocnemius muscle of SOD1^G93A^ mice increased significantly over disease progression, peaking at the age of 16 weeks (Figures 2B,C). At this time point, the RNA levels of *lpar2* in both tissues were ~2.5 fold higher in SOD1^G93A^ mice relative to wildtype littermates (Figures 2B,C).

### Genetic Deletion of Lpar2 Modifies ALS Disease Course

**Figure 3:**
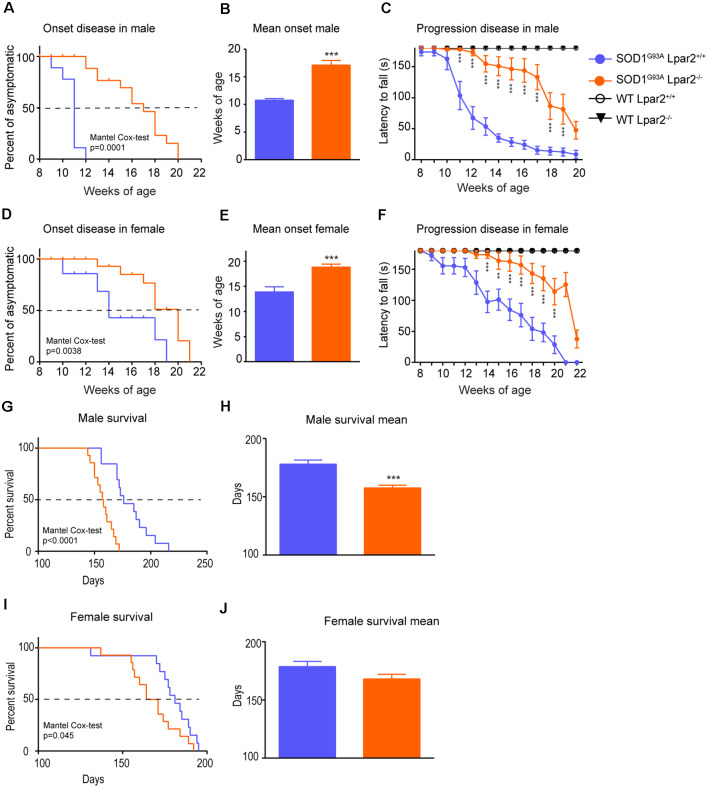
Dual effects of LPA_2_ in ALS disease. **(A–E)** Graphs showing the functional evaluation of ALS mice in the rotarod test over time. **(A,B)** *Lpar2* deficiency delays the onset of the disease in 5 weeks in male ALS mice **(A,B)** and 4 weeks in females **(D,E)**. Graphs showing the functional progression of neurological deficits represented by the latency to fall off the rotarod test in males **(C)** and females **(F)**. **(G–J)** Effects of *Lpar2* gene deletion in the lifespan of ALS mice. Two-way RM ANOVA, Bonferroni’s *post hoc* test in **(A,B)**. Mantel-Cox test in **(B,E,G,I)**. Unpaired *t*-test in **(C,F,H,J)**. **(A–C)** Males (*n* = 9 SOD1^G93A^ *Lpar2*^+/+^, *n* = 11 SOD1^G93A^ *Lpar2*^−/−^, *n* = 4 WT *Lpar2*^+/+^, *n* = 4 WT Lpar2^−/−^). **(D–E)** Females (*n* = 10 SOD1^G93A^ *Lpar2*^+/+^, *n* = 11 SOD1^G93A^ *Lpar2*^−/−^, *n* = 4 WT *Lpar2*^+/+^, *n* = 4 WT *Lpar2*^−/−^). **(G–H)** Males (*n* = 14 SOD1^G93A^ *Lpar2*^+/+^, *n* = 15 SOD1^G93A^ *Lpar2*^−/−^). **(I–J)** Females (*n* = 14 SOD1^G93A^ *Lpar2*^+/+^, *n* = 14 SOD1^G93A^ *Lpar2*^−/−^). **p < 0.01, ***p < 0.001, vs. SOD1^G93A^ LPA_2_^+/+^. Data are shown as mean ± SEM.

To determine the contribution of LPA_2_ in ALS, we crossed SOD1^G93A^ mice with *Lpar2* deficient mice (SOD1^G93A^-*Lpar2*^−/−^). We observed that the absence of *Lpar2* significantly delayed ALS disease onset by 4 and 5 weeks in female and male mice, respectively (Figures 3A–F). In this line, the rotarod test also revealed that the genetic deletion of *Lpar_2_* markedly slowed the neurological decline of ALS mice (Figures 3A,D). Unexpectedly, the lack of *Lpar2* did not increase the lifespan of SOD1^G93A^, despite its absence markedly protected against the clinical signs of the disease (Figures 3G–J). Indeed, deficiency of *Lpar2* shortened the lifespan of ALS mice by 21 and 10 days, in males and females, respectively (Figures 3H,J). Therefore, these observations suggest that the lack of LPA_2_ signaling slows disease onset and progression but shortens survival.

### LPA_2_ Does Not Contribute to Motoneuron Death in ALS

**Figure 4:**
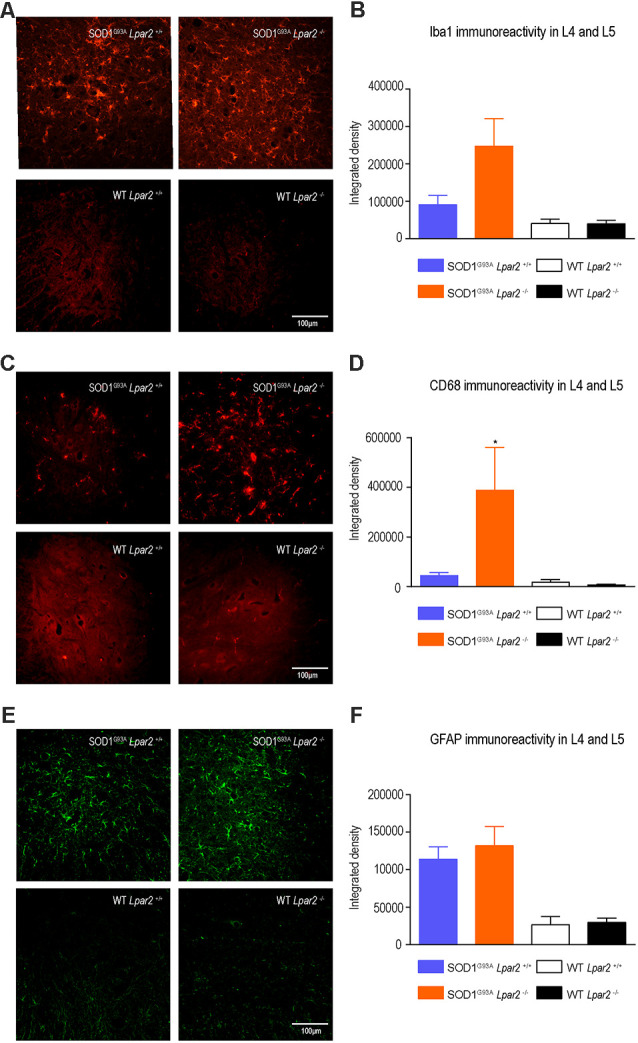
The lack of *Lpar2* does not reduce astrogliosis and microgliosis in the lumbar spinal cord of SOD1^G93A^ mice. **(A)** Representative images of the lumbar spinal cord stained against Iba1. **(B)** Plot showing the quantification of the Iba1 immunoreactivity in the L4 and L5 spinal cord segments. **(C)** Representative images of the lumbar spinal cord showing stained against CD68. **(D)** Graph showing quantification of CD68 immunoreactivity in the L4 and L5 segments. **(E)** Representative images of the lumbar spinal cord showing stained against GFAP. **(F)** Graph showing quantification of GFAP immunoreactivity in the L4 and L5 segments. One-way ANOVA, Bonferroni’s *post hoc* test. *p < 0.05 vs. SOD1^G93A^ LPA_2_^+/+^ (males; *n* = 3 SOD1^G93A^ *Lpar2*^+/+^, *n* = 4 SOD1^G93A^ *Lpar2*^−/−^, *n* = 4 WT *Lpar2*^+/+^, *n* = 4 WT *Lpar2*^−/−^). Data are shown as mean ± SEM.

To gain insights into the early detrimental actions of LPA_2_ in ALS, we focused our experiments on SOD1^G93A^ mice at 16 weeks of age, when neurological outcomes were markedly enhanced in ALS mice lacking *lpar2*. Since LPA mediates gliosis and we recently reported that microglial cells become cytotoxic upon LPA_2_ stimulation (López-Serrano et al., 2019), we first evaluated whether LPA_2_ signaling contributed to microgliosis and astrogliosis in the lumbar spinal cord of SOD1^G93A^ mice. Histological analysis revealed that immunoreactivity for Iba1, a marker for microglial cells, tended to be increased in the spinal cord ventral horn of ALS mice deficient in *Lpar2*, although this result did not reach statistical significance (Figures 4A,B). However, CD68 expression in microglial cells, which is considered a marker of activated cells, was significantly increased in ALS mice deficient in *Lpar2* (Figures 4C,D), suggesting that although the lack of Lpar2 signaling did not increase microgliosis it resulted in greater activation of this glial cell subset. Immunoreactivity for GFAP, a marker for astrocytes, did not differ in the spinal cord of both ALS experimental groups (Figures 4E,F).

**Figure 5:**
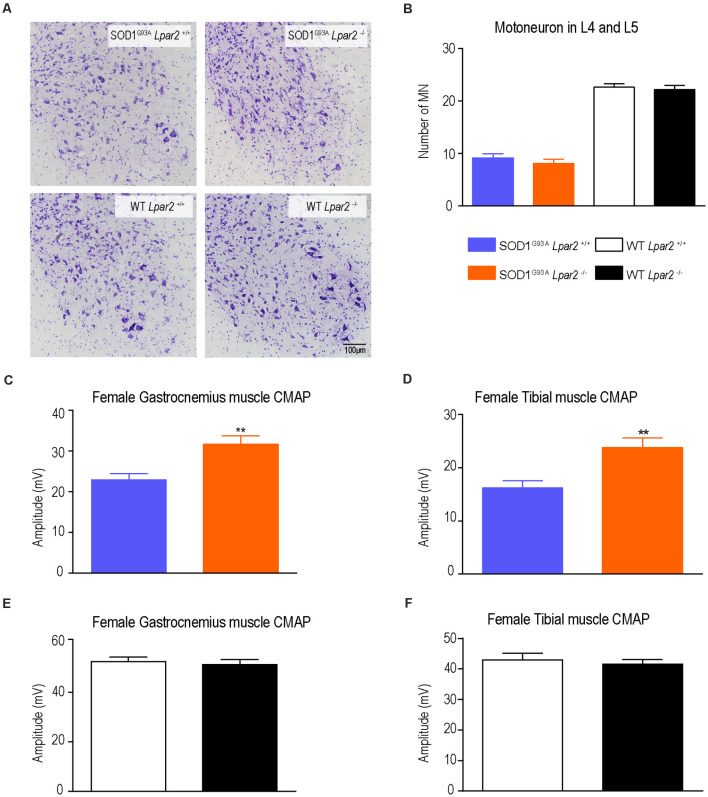
The lack of Lpar2 does not increase motoneuron survival but preserves neuromuscular integrity in ALS mice at 16 weeks of age. **(A)** Representative micrograph of lumbar spinal cord showing motoneurons stained with cresyl violet. **(B)** Quantification of motoneurons in the L4 and L5 spinal cord segments (males; *n* = 6 SOD1^G93A^ *Lpar2*^+/+^, *n* = 9 SOD1^G93A^ *Lpar2*^−/−^, *n* = 7 WT *Lpar2*^+/+^, *n* = 8 WT *Lpar2*^−/−^). **(C,D)** Plots showing the compound muscle potential amplitude of the gastrocnemius **(C,E)** and tibialis anterior **(D,F)** muscle at 16 weeks of age (males; *n* = 14 SOD1^G93A^ *Lpar2*^+/+^, *n* = 14 SOD1^G93A^ *Lpar2*^−/−^, *n* = 5 WT *Lpar2*^+/+^, *n* = 5 WT *Lpar2*^−/−^). **p < 0.01 vs. SOD1^G93A^ LPA_2_^+/+^. One-way ANOVA, Bonferroni’s *post hoc* test. Data are shown as mean ± SEM.

Since degeneration of motoneurons is the main feature in ALS mice and patients, we assessed whether LPA_2_ contributed to motoneuron death. Histopathological analysis of the lumbar spinal cord (L4 and L5) revealed that the lack of *Lpar2* did not have any effect on motoneuron survival in ALS mice (Figures 5A,B) despite electrophysiological tests revealing that the amplitude of the CMAPs was significantly increased in ALS mice deficient in *Lpar2* at 16 weeks of age (Figures 5C–F). These results indicated that neuromuscular integrity was enhanced in SOD1^G93A^ mice lacking *Lpar2* but this was not due to detrimental actions of LPA_2_ on motoneuron. These observations, therefore, suggest that the initial harmful effects of LPA_2_ in ALS may be mediated in the peripheral nerve and/or skeletal muscle.

### LPA_2_ Does Not Promote Myelin Damage in Motor Axons

**Figure 6:**
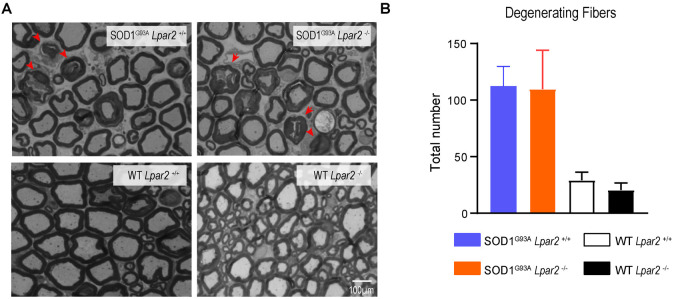
*Lpar2* deletion does alter Wallerian degeneration of motor axons in SOD1^G93A^ mice. **(A)** Representative cross-section images of the spinal nerve ventral root (L4) stained in toluidine blue. Note the presence of fiber with disrupted myelin sheaths the motor roots of ALS mice (arrowhead). **(B)** Graph showing the quantification of fibers with some signs of myelin degeneration at 16 weeks of age in male mice (*n* = 3 SOD1^G93A^ *Lpar2*^−/−^, *n* = 5 SOD1^G93A^ *Lpar2*^+/+^, *n* = 4 WT LPA_2_^−/−^, *n* = 3 WT *Lpar2*^+/+^). Two-way ANOVA, Bonferroni’s *post hoc* test. Data are presented as mean ± SEM.

LPA contributes to demyelination in the spinal cord by signaling via LPA_1_ (Santos-Nogueira et al., 2015) and LPA_2_ (López-Serrano et al., 2019). Considering that *Lpar2* transcripts peaked in the sciatic nerve of ALS mice at 16 weeks of age, we assessed whether LPA_2_ led to demyelination and/or degeneration of motor axons. For this purpose, we quantified the number of fibers that showed some signs of Wallerian degeneration. These analyses revealed that the counts of motor fibers that showed signs of myelin breakdown did not differ between both ALS groups (Figures 6A,B). These results, therefore, reveal that LPA_2_ does not promote demyelination in ALS.

### The Absence of *Lpar2* Prevents Muscle Atrophy in SOD1^G93A^ Mice

**Figure 7:**
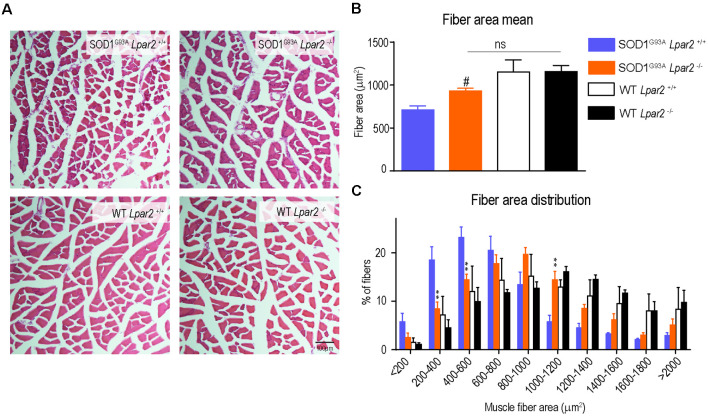
*Lpar2* gene deletion prevents muscle atrophy in SOD1^G93A^ mice. **(A)** Representative images of gastrocnemius muscle stained in hematoxylin and eosin at 16 weeks of age in male mice. **(B)** Quantification of the muscle cross-section area at 16 weeks of age in male mice. **(C)** Plot showing the distribution of muscle fiber width (males; *n* = 4 per group). One-way ANOVA, Bonferroni’s *post hoc* test. ^#^*p* < 0.05 vs. WT *Lpar2*^+/+^; ns, not significant vs. WT *Lpar2*^−/−^ in **(B)**; Two-way ANOVA, Bonferroni’s *post hoc* test in **(C)**. **p < 0.01 vs. SOD1^G93A^ *Lpar2*^+/+^. Data are presented as mean ± SEM.

Since the data reported above suggested that initial detrimental actions of LPA_2_ in SOD1^G93A^ mice were unlikely to be mediated in the spinal cord or peripheral nerves, we then studied the effects of *Lpar2* deficiency in the muscle of ALS animals. Highlight that *lpar2* transcripts in the gastrocnemius muscle of SOD1^G93A^ mice peaked at the age of 16 weeks (Figure 2C). We found that cross tissue sections of the gastrocnemius muscle stained with hematoxylin and eosin revealed that the mean muscle fiber area was decreased in SOD1^G93A^ mice as compared to wildtype mice, indicating clear signs of muscle atrophy (Figures 7A,B). The mean muscle fiber area was slightly enhanced in ALS mice lacking *Lpar2*, although this result did not reach statistical significance (Figures 7A,B). However, further analysis revealed that the distribution of the muscle fiber according to their size was shifted towards the right in ALS mice deficient for *Lpar2*. These data indicated that the proportion of fibers with larger diameter was increased in ALS lacking *lpar2*, whereas SOD1^G93A^ *Lpar2*^+/+^ animals displayed a greater proportion of small muscle fibers (Figure 7C). These data, therefore, indicate that LPA_2_ signaling contributes to muscle atrophy in ALS.

**Figure 8:**
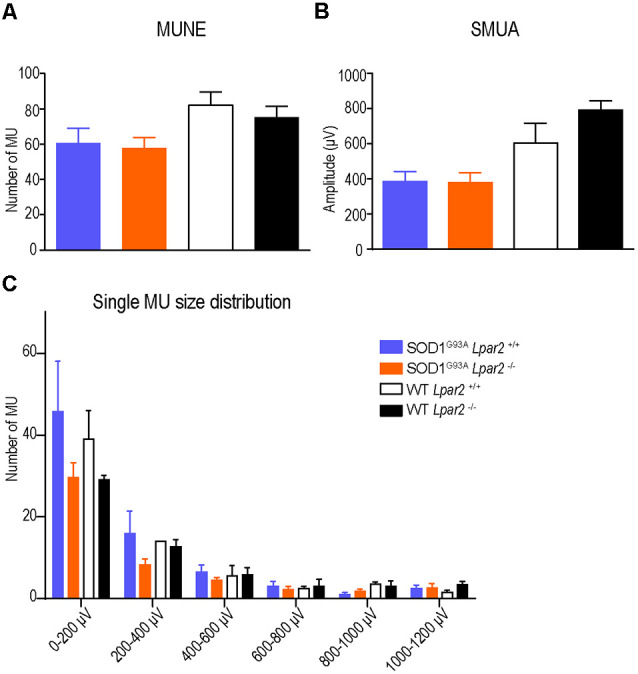
The absence of *Lpar2* does not enhance collateral sprouting. **(A–C)** Electrophysiological estimation of motor unit number **(A)**, mean amplitude of single motor unit potential **(B)**, and single size of motor units **(C)** of the gastrocnemius muscle at 16 weeks of age in male mice. Note that the absence of *Lpar2* does not modify any of these parameters (males; *n* = 5 SOD1^G93A^ *Lpar2*^−/−^, *n* = 8 SOD1^G93A^ *Lpar2*^+/+^, *n* = 5 WT *Lpar2*^−/−^, *n* = 3 WT *Lpar2*^+/+^). One-way ANOVA with Bonferroni’s *post hoc* test in **(A,B)**; Two-way RM ANOVA with Bonferroni’s *post hoc* test in **(C)**. Data shown as mean ± SEM.

To determine whether protection against muscle atrophy observed in ALS mice deficient in *Lpar2* was due to increased collateral sprouting of motor axons, we assessed the integrity of the neuromuscular junctions (NMJ) in the gastrocnemius muscle. Electrophysiological estimation of the motor unit number (MUNE), the mean amplitude of single motor unit potential (SMUA), and the distribution of motor unit action potential (MUAP) amplitude revealed that enhanced collateral sprouting was not the mechanism that prevented muscle atrophy in ALS mice lacking *Lpar2* (Figure 8).

**Figure 9:**
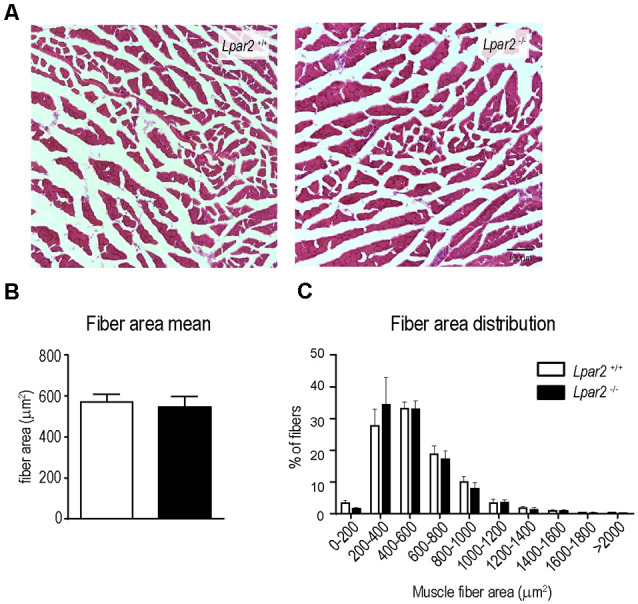
*Lpar2* deficiency does not prevent muscle atrophy after sciatic nerve injury. **(A)** Representative images of gastrocnemius muscle cross tissue sections at 21 days after sciatic nerve transection. **(B,C)** Plot showing the quantification of the muscle cross-section area **(B)** and the distribution of muscle fiber width **(C)**. *Lpar2*^−/−^ mice (*n* = 4) *Lpar2*^+/+^ mice (*n* = 6). Unpaired *t*-student in **(B)**; Two-way RM ANOVA with Bonferroni’s *post hoc* test in **(C)**. Data shown as mean ± SEM.

Previous work revealed that muscle was a primary target of mutant SOD1 protein (SOD1^G93A^) and that selective expression of SOD1^G93A^ in skeletal muscle cells induced atrophy and functional impairments (Dobrowolny et al., 2008). Therefore, the attenuation of muscle atrophy and neurological decline observed in ALS mice lacking *Lpar2* could be due to the deleterious actions of this LPA receptor on innervated muscle fibers. However, the possibility that LPA_2_ signaling could be required for the atrophy of denervated muscle fibers cannot be discarded. To dissect out whether LPA_2_ signaling is crucial for the atrophy of denervated fibers, we transected the sciatic nerve of *Lpar2* knockout mice and wildtype littermates to promote muscle denervation and ligated the injured nerve to prevent axon regeneration and muscle reinnervation. Histological cross-sections of gastrocnemius muscles harvested at 21 after lesion revealed that the lack of *Lpar*2 signaling did not alter muscle atrophy caused by the denervation of the nerve (Figures 9A–C). These data, therefore, indicated that LPA_2_ is not required for the atrophy of denervated fiber, and suggested, that the reduced atrophy observed in ALS mice lacking *Lpar2* was due to greater protection of innervated muscle fibers.

**Figure 10:**
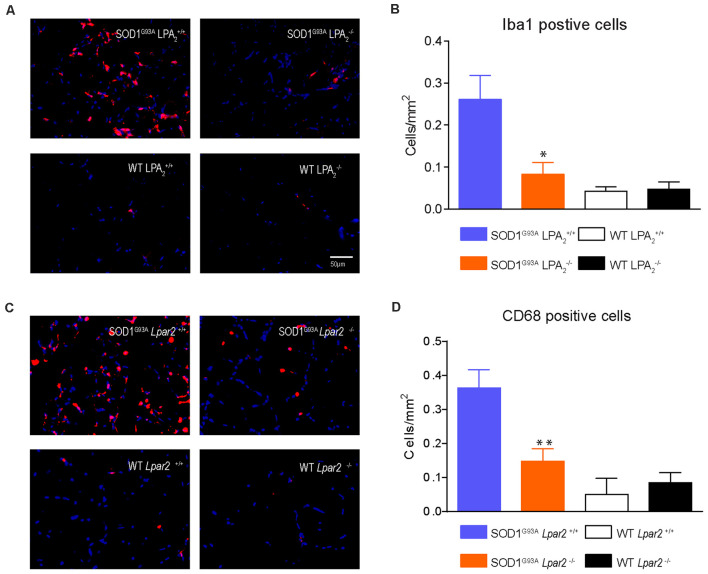
Gene deletion of *Lpar2* reduces macrophage invasion in the muscle of ALS mice. **(A)** Representative images of gastrocnemius muscle cross tissue sections stained against Iba1. **(B)** Plot showing the quantification of the Iba1+ cells. **(C)** Representative images of gastrocnemius muscle cross tissue sections stained against CD68. **(D)** Plot showing the quantification of the CD68+ cells. *n* = 4 per group. One-way ANOVA with Bonferroni’s *post hoc* test. *p < 0.05, **p < 0.01 vs. SOD1^G93A^ *Lpar2*^+/+^. Data are presented as mean ± SEM.

Recent studies reveal that immune cells infiltrate into the muscles of SOD1^G93A^ animals and that they contribute to disease progression (Van Dyke et al., 2016; Trias et al., 2017). Since LPA is involved in triggering inflammation (Choi and Chun, 2013; Yung et al., 2014) we assessed whether LPA_2_ signaling is involved in regulating muscle inflammation in ALS. Histological analysis of gastrocnemius muscle showed that the number of macrophages (Iba1+ cells) was significantly reduced in SOD1^G93A^ mice lacking *Lpar2* (Figures 10A,B). In this line, the expression of CD68, which is found in activated macrophages, was also reduced in the muscles of ALS mice deficient in Lpar2 (Figures 10C,D), suggesting that LPA_2_ signaling contributes to muscle inflammation in ALS.

### Discussion

In the present study, we assessed the effects of LPA_2_ on ALS. We showed that *LPA_2_* mRNA levels are increased in post-mortem spinal cord samples of ALS patients, as well as in the sciatic nerve and gastrocnemius muscle of ALS mice. We also identified a biphasic role of LPA_2_ in ALS. Initially, LPA_2_ signaling accelerates disease onset and neurological deficits, whereas later, LPA_2_ extends survival. In the search for the mechanisms underlying the harmful actions of LPA_2_ at the early stages of ALS disease, we revealed this receptor did not promote spinal cord gliosis, motoneuron death, motor axon demyelination, but it increased muscle atrophy and inflammation. Overall, this work provides, for the first time, evidence for the involvement of LPA_2_ in ALS physiopathology.

Inflammation is a hallmark of neurodegenerative diseases, typified by reactive morphology of astrocytes and microglia, accompanied by the release of inflammatory mediators in the parenchyma (Ransohoff, 2016). This physiological response has been shown in ALS in both patients and animal models (Philips and Robberecht, 2011), as well as, in most neurological conditions including Alzheimer’s, Parkinson and frontotemporal lobe dementia among others (De Virgilio et al., 2016; Newcombe et al., 2018). Although most of the ALS reports have focused on studying the inflammatory response in the CNS, it is currently known that in ALS there is also an invasion of immune cells into the peripheral nerves and muscles that contributes to disease progression (Martínez-Muriana et al., 2016; Van Dyke et al., 2016; Trias et al., 2017; Trias et al., 2018, 2020).

Studies done over the last years have demonstrated that lipids play a crucial role in the regulation of the inflammatory response. Among them, lipids, such as the lysophospholipids (LPLs; Chun et al., 2014), have demonstrated inflammatory effects that are both detrimental and beneficial, including within the CNS (David et al., 2012). LPA is an LPL that acts as an extracellular and intracellular signaling molecule and controls a wide range of physiological responses. The effects of LPA are mediated by 6 G protein-coupled receptors, LPA_1_–LPA_6_ (Choi and Chun, 2013; Kihara et al., 2014; Yung et al., 2015). Earlier studies demonstrated that this bioactive lipid has important actions in the CNS after injury and disease. LPA activates astrocytes and microglia, causes axonal retraction, induces brain blood barrier permeability among others (Sorensen et al., 2003; Choi et al., 2010). We previously reported that LPA_1_ and LPA_2_ that are normally associated with myelination (Weiner et al., 1998, 2001; Weiner and Chun, 1999; Anliker et al., 2013) also cause demyelination and functional impairments after spinal cord injury, in part, by activating microglial cells (Santos-Nogueira et al., 2015; López-Serrano et al., 2019).

In the present work, we found that *LPA_1_* and *LPA_2_* RNA transcripts were expressed in post-mortem human spinal cord samples and that the transcripts of LPA_2_, but not LPA_1_, were increased ~2 fold in sALS patients. The expression of *Lpar2* in the spinal cord of SOD1^G93A^ mice tended to increase once the clinical signs of the disease were evident, although this up-regulation did not reach statistical significance. Since ALS mouse samples were not harvested at the disease endpoint, unlike the spinal cords of sALS patients, this may account for the lower *Lpar2* transcript levels observed in the ALS animal model. We also found that *Lpar2* RNA levels were up-regulated in the sciatic nerve and the gastrocnemius muscle of ALS mice, both peaking at 16 weeks of age. The source of LPA_2_ in ALS was not studied due to the lack of specificity of current antibodies. However, *Lpar2* transcripts in the CNS have been described in neurons, oligodendrocytes, microglia, and infiltrating macrophages, whereas in the peripheral nervous system and skeletal muscle have been shown in Schwann cells and muscle fibers, respectively (Frohnert et al., 2003; Jean-Baptiste et al., 2005; Zhang et al., 2014).

To assess whether LPA_2_ signaling contributes to ALS, we crossed mice lacking *Lpar2* with SOD1^G93A^ mice. This double-transgenic animal revealed for the first time that the absence of *Lpar2* delayed the onset of ALS disease in both males and females, and slowed the deterioration of locomotor performance. Despite the detrimental actions of LPA_2_ at the initial stages of the disease, deficiency in *Lpar2* shortened the lifespan of SOD1^G93A^ mice. These data suggest that LPA_2_ signaling has a dual role in ALS, being detrimental at the early stages of the disease and later, being protective. However, we do not discard that LPA_2_ signaling could play an important physiological role for neuronal function, and its genetic removal could increase their vulnerability to stress conditions. Therefore, the development of LPA_2_ agonist is critical to elucidate whether activation of this receptor confers protection at late stages of ALS disease. Highlight that lifespan of SOD1^G93A^ mice was about 2 weeks increased than that reported from Jackson Laboratories. This could be due to the background acquired by ALS mice after being bred with the LPA_2_ mice. However, other factors related to the loss of SOD1^G93A^ gene copies or the housing conditions of the animal facility, cannot be discarded.

To elucidate the early pathological role of LPA_2_ in ALS, we assessed its effects at different levels in which motor function is involved (spinal cord, peripheral nerve, and muscle). We first focused on the effects of LPA_2_ in the spinal cord of ALS animals, where lower motoneurons are located (Contos et al., 2002; Mancuso et al., 2014; McCampbell et al., 2018). Despite having previously reported that activation of LPA_2_ exerts deleterious effects after spinal cord injury (López-Serrano et al., 2019), we observed that the lack of *Lpar2* did not modify the number of motoneurons in ALS mice, suggesting that the harmful effects of LPA_2_ in ALS were independent on motoneuron death. We also found that the lack of *Lpar2* did not attenuate astrogliosis but increase the activation of microglial cells in the lumbar spinal cord of ALS mice, as revealed the CD68 expression. Although microglia and astroglia activation at later stages of the disease was not studied in the present work, the increased microglia reactivity observed in ALS mice deficient in *Lpar2* might indicate that this receptor could put the brakes on microglia activation in advanced stages of the disease, which may account for the shorter survival of ALS mice lacking *Lpar2*.

In ALS, axonopathy precedes and underlies skeletal muscle weakness and progressive paralysis. Indeed, peripheral axons degenerate before the death of cell bodies in the CNS, according to evidence in ALS patients and murine models (Fischer and Glass, 2007; Moloney et al., 2014; Trias et al., 2020). Unlike peripheral nerve injury, axonal growth of degenerated axons is limited in ALS, leading to chronic and progressive axon degeneration, recruitment of immune cells, and promotion of inflammation (Trias et al., 2020). In this line, treatment with a CSF1R antagonist attenuated the influx of macrophages into the nerves of ALS mice, that together with its effects on minimizing microgliosis, extended mice survival (Martínez-Muriana et al., 2016). Little is known about the specific functions of LPA_2_ in the sciatic nerve, but similar to LPA_1_, this receptor is found in Schwann cells and its expression appears shortly before maturation/myelination (Weiner and Chun, 1999; Weiner et al., 2001; García-Díaz et al., 2015). Indeed, activation of LPA_2_ in myelinating cells seems to play a key role in myelin formation (Weiner and Chun, 1999; Weiner et al., 2001; Anliker et al., 2013; García-Díaz et al., 2015).

Previous works revealed that LPA_2_ activation contributes to demyelination after spinal cord injury (Santos-Nogueira et al., 2015; López-Serrano et al., 2019) suggesting that the upregulation of *Lpar2* in the peripheral nerves of ALS mice could mediate demyelination and/or damage in motor axons. However, histological analysis of the spinal cord motor roots of ALS mice, where motor axons are present, revealed that the absence of *Lpar2* did not prevent degeneration of the axons or their myelin sheaths, and suggested that the detrimental actions of LPA_2_ in ALS could be mediated in the skeletal muscle.

Skeletal muscle atrophy in ALS has been interpreted as a consequence of degeneration of motor axons. Nevertheless, it has been recently postulated that skeletal myocytes also have an active role in muscle atrophy in ALS, independent from motoneuron loss. This is clear from experiments using mice with selective expression of SOD1^G93A^ in muscle cells, which revealed that skeletal muscle is a primary target of SOD1^G93A^-mediated toxicity and causes progressive muscle atrophy (Dobrowolny et al., 2008). In this view, numerous studies have attempted to palliate muscle pathology to counterbalance the effects of motoneuron degeneration (Derave et al., 2003; Gifondorwa et al., 2007; Yoo and Ko, 2012; Halon et al., 2014; Loeffler et al., 2016). Here, we observed that *Lpar2* deficiency protected from muscle atrophy in ALS animals.

Collateral axonal sprouting is a natural mechanism observed in ALS disease and nerve injury, among other disorders, in which surviving motoneurons reinnervate denervated end-plates within the same muscle, enabling to compensate for the loss of functional motor units and prevent muscle atrophy (Siu and Gordon, 2003). However, we revealed using electrophysiological techniques that deficiency in *Lpar2* did not enhance collateral axonal sprouting in ALS. We also found that LPA_2_ signaling was not required for the events that lead to atrophy of denervated muscle fibers, since muscle atrophy caused by sciatic nerve injury was not altered in *Lpar2* null mice. These observations suggested that LPA_2_ signaling contributed to skeletal muscle atrophy of ALS mice by promoting deterioration of innervated muscle fibers.

A recent study revealed that administration of LPA after muscle injury worsens atrophy (Davies et al., 2017). The same work demonstrated that the detrimental effects of LPA in muscle injury were likely due to the actions of LPA on inflammation since this biolipid increased the expression of pro-inflammatory cytokines in the muscle and increased the invasion of immune cells (Davies et al., 2017). As reported above, mutant SOD1 protein causes a direct toxic effect in the skeletal muscle cell and leads to muscle atrophy (Dobrowolny et al., 2008). It is now known that different immune cell subsets infiltrate the skeletal muscle of ALS animals, which is likely due to the damage of muscle fibers, and contribute to disease pathogenesis (Van Dyke et al., 2016; Trias et al., 2018). Interestingly, we observed that the infiltration of macrophages in skeletal muscles of SOD1^G93A^ mice was markedly attenuated in the lack of *Lpar2*. These findings suggest that muscle damage caused by mutant SOD1 favors the recruitment of macrophages into the muscle, in part, by LPA_2_-dependent mechanisms, and causes further muscle deterioration, and consequently, motor function decline.

To summarize, this work provides the first evidence that LPA_2_ signaling contributes to ALS. Initial activation of LPA_2_ accelerates ALS progression by promoting muscle atrophy, which is likely due to the inflammatory actions of this receptor in the muscle, whereas later, it promotes beneficial effects on survival.

## Data Availability Statement

The raw data supporting the conclusions of this article will be made available by the authors, without undue reservation.

## Ethics Statement

The studies involving human participants were reviewed and approved by the Clinical Research Ethics Committee (CEIC) of the Bellvitge University Hospital. The patients/participants provided their written informed consent to participate in this study. The animal study was reviewed and approved by Animal Experimentation Ethical Committee of the Universitat Autónoma de Barcelona (CEEAH 1188R3-DMAH 3131)/(2969).

## Author Contributions

RL-V and MP-P designed the study. MP-P, AM-M, PA-B, IF, and RL-V performed the research, analyzed, or interpreted results. JC created LPA_2_ mutant mice. MP-P, JC, and RL-V wrote the manuscript. All authors contributed to the article and approved the submitted version.

## Conflict of Interest

The authors declare that the research was conducted in the absence of any commercial or financial relationships that could be construed as a potential conflict of interest.
